# High-Sensitivity Cardiac Troponin Publications during the COVID-19 Pandemic (2020–2022)

**DOI:** 10.3390/jcdd10010005

**Published:** 2022-12-23

**Authors:** Peter A. Kavsak

**Affiliations:** Department of Pathology and Molecular Medicine, McMaster University, Hamilton, ON L8S 4K1, Canada; kavsakp@mcmaster.ca

The first publications detailing the clinical utility of high-sensitivity cardiac troponin (hs-cTn) in patients with possible acute coronary syndrome (ACS) are traceable to 2009 [1,2,3]. In early 2020, I wrote an editorial where I concluded that, “before we obtain 20/20 vision for hs-cTn testing in and outside the ACS setting additional publications and data from all assays will be needed” [3]. This publication coincided with the commencement of the COVID-19 pandemic and, surprisingly, the acceleration of publications with hs-cTn in and outside the ACS setting, with the majority of manufacturers and their hs-cTn assays (Figure 1).

The COVID-19 pandemic has not only increased the number of scientific articles, but also the impact factor for journals, which is a testament to the scientific community’s response to the pandemic [4]. Regarding cardiac biomarkers and laboratory tests, early in the course of the pandemic, data was emerging regarding possible cardiac complications and COVID-19 [5,6]. In particular, some younger patients that were not hospitalized for COVID-19 may develop myocardial injury during their recovery [6]. The role of an objective biomarker here, such as hs-cTn, is extremely valuable; albeit not all hs-cTn elevations following COVID-19 infection or immunization against the virus reflect ongoing, acute injury [7]. Macrocomplexes may be a possible culprit for some of these elevations in some patients in this setting, and outside of COVID-19, macrocomplexes and other new interferences as well as different combinations of known (pre-analytical) variables may result in discordant hs-cTn levels as highlighted in articles published in the past couple of years [7,8,9,10,11,12].

The impact of interferences resulting in falsely elevated hs-cTn concentrations is not trivial, with reports for one manufacturer’s assay yielding not only a higher prevalence of myocardial injury due to the presence of an interference but also a higher rate of difficult-to-reproduce results (i.e., high imprecision) [10]. The latter finding of poor reproducibly is terribly important as many guidelines have recommended pathways that incorporate a change in hs-cTn concentrations to either rule-out or rule-in myocardial infarction (MI) in patients with symptoms suggestive of ACS [13,14]. To that end, the clinical laboratory needs to provide a highly precise and a robust hs-cTn assay or pathway, to prevent patient misclassification due to suboptimal analytical variation or the presence of interferences impacting measurements. Even here, not all pathways will achieve the same level of diagnostic performance [14,15]. Specifically, in an undifferentiated emergency department (ED) patient population being evaluated with hs-cTn, none of the pathways/algorithms (e.g., the European Society of Cardiology, High-STEACS, COMPASS-MI) which use hs-cTn alone achieved a sensitivity ≥99% for MI or death at thirty days following the ED presentation [15]. However, algorithms incorporating additional laboratory tests (i.e., troponin plus other laboratory tests), can achieve the 99% sensitivity benchmark [16], with the application of these laboratory-based algorithms yielding higher sensitivity estimates for thirty day MI or death as compared to hs-cTn alone as evident in very large ED population datasets [17,18].

Outside of COVID-19 and the ED setting, publications have also identified unique aspects pertinent for hs-cTn. For example, in patients undergoing cardiac surgery pre-operative and post-operative hs-cTn measurements may provide clinical utility [19,20]. Importantly, levels of hs-cTn greater than 200 times the upper reference limit within one day after cardiac surgery are prognostic, which is a much higher level than what is stated in the universal definition of myocardial infarction [20,21]. In fact, the utility of hs-cTn extends beyond these settings with possible important roles in cancer therapy related cardiotoxicity, in heart failure and other supply-demand mismatch situations [22,23,24,25].

When my 2020 editorial was available online (25 January 2020); “The WHO Regional Director for Europe issued a public statement outlining the importance of being ready at the local and national levels for detecting cases, testing samples and clinical management.” https://www.who.int/emergencies/diseases/novel-coronavirus-2019/interactive-timeline#! (accessed on 11 December 2022). The exponential growth of SARS-CoV-2 infection did not equate to the exponential growth of publications related to hs-cTn (see Figure 1). However, the increased number of publications related to hs-cTn during this time has indeed diminished the knowledge gap as outlined in the 2020 editorial and even opened the door to new approaches and pathways for this important laboratory test.

## Figures and Tables

**Figure 1:**
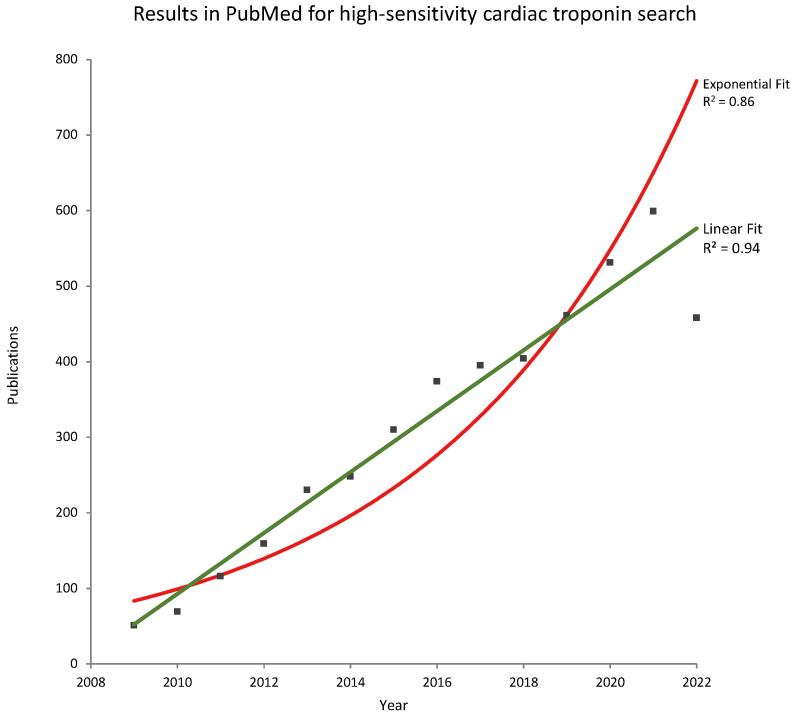
Publications listed per year in PubMed for the search term high-sensitivity cardiac troponin from 2009 to 2022 (search date 9 December 2022).

## References

[1] Kavsak P.A., MacRae A.R., Yerna M.J., Jaffe A.S. (2009). Analytic and clinical utility of a next-generation, highly sensitive cardiac troponin I assay for early detection of myocardial injury. Clin. Chem..

[2] Reichlin T., Hochholzer W., Bassetti S., Steuer S., Stelzig C., Hartwiger S., Biedert S., Schaub N., Buerge C., Potocki M. (2009). Early diagnosis of myocardial infarction with sensitive cardiac troponin assays. N. Engl. J. Med..

[3] Kavsak P.A. (2020). Approaching 2020 acuity for high-sensitivity cardiac troponin assays in Clinical Biochemistry. Clin. Biochem..

[4] Lippi G., Plebani M. (2022). Clinical Chemistry and Laboratory Medicine: Enjoying the present and assessing the future. Clin. Chem. Lab. Med..

[5] Kavsak P.A., Hammarsten O., Worster A., Smith S.W., Apple F.S. (2021). Cardiac Troponin Testing in Patients with COVID-19: A Strategy for Testing and Reporting Results. Clin. Chem..

[6] Mohamed Ali A., Wasim D., Larsen T.H., Bogale N., Bleie Ø., Saeed S. (2021). Acute Myocardial Infarction Due to Microvascular Obstruction in a Young Woman Who Recently Recovered from COVID-19 Infection. J. Cardiovasc. Dev. Dis..

[7] Bularga A., Oskoui E., Fujisawa T., Jenks S., Sutherland R., Apple F.S., Hammarsten O., Mills N.L. (2022). Macrotroponin Complex as a Cause for Cardiac Troponin Increase after COVID-19 Vaccination and Infection. Clin. Chem..

[8] Harley K., Bissonnette S., Inzitari R., Schulz K., Apple F.S., Kavsak P.A., Gunsolus I.L. (2021). Independent and combined effects of biotin and hemolysis on high-sensitivity cardiac troponin assays. Clin. Chem. Lab. Med..

[9] Kavsak P.A., Clark L., Martin J., Mark C.T., Paré G., Mondoux S., Chetty V.T., Ainsworth C., Worster A. (2021). Acute Phase Response and Non-Reproducible Elevated Concentrations with a High-Sensitivity Cardiac Troponin I Assay. J. Clin. Med..

[10] Kavsak P.A., Mondoux S.E., Martin J., Hewitt M.K., Clark L., Caruso N., Mark C.T., Chetty V.T., Ainsworth C., Worster A. (2021). Disagreement between Cardiac Troponin Tests Yielding a Higher Incidence of Myocardial Injury in the Emergency Setting. J. Cardiovasc. Dev. Dis..

[11] Lafrenière M.A., Tandon V., Ainsworth C., Nouri K., Mondoux S.E., Worster A., Kavsak P.A. Storage conditions, sample integrity, interferences, and a decision tool for investigating unusual high-sensitivity cardiac troponin results. Clin. Biochem..

[12] Lam L., Tse R., Gladding P., Kyle C. (2022). Effect of Macrotroponin in a Cohort of Community Patients with Elevated Cardiac Troponin. Clin. Chem..

[13] Collet J.P., Thiele H., Barbato E., Barthélémy O., Bauersachs J., Bhatt D.L., Dendale P., Dorobantu M., Edvardsen T., Folliguet T. (2021). 2020 ESC Guidelines for the management of acute coronary syndromes in patients presenting without persistent ST-segment elevation. Eur. Heart J..

[14] Chiang C.H., Chiang C.H., Pickering J.W., Stoyanov K.M., Chew D.P., Neumann J.T., Ojeda F., Sörensen N.A., Su K.Y., Kavsak P. (2022). Performance of the European Society of Cardiology 0/1-Hour, 0/2-Hour, and 0/3-Hour Algorithms for Rapid Triage of Acute Myocardial Infarction: An International Collaborative Meta-analysis. Ann. Intern. Med..

[15] Kavsak P.A., Hewitt M.K., Mondoux S.E., Cerasuolo J.O., Ma J., Clayton N., McQueen M., Griffith L.E., Perez R., Seow H. (2021). Diagnostic Performance of Serial High-Sensitivity Cardiac Troponin Measurements in the Emergency Setting. J. Cardiovasc. Dev. Dis..

[16] Kavsak P.A., Mondoux S.E., Hewitt M.K., Ainsworth C., Hill S., Worster A. (2021). Can the Addition of NT-proBNP and Glucose Measurements Improve the Prognostication of High-Sensitivity Cardiac Troponin Measurements for Patients with Suspected Acute Coronary Syndrome?. J. Cardiovasc. Dev. Dis..

[17] Kavsak P.A., Cerasuolo J.O., Ko D.T., Ma J., Sherbino J., Mondoux S.E., Perez R., Seow H., Worster A. (2020). High-Sensitivity Cardiac Troponin I vs a Clinical Chemistry Score for Predicting All-Cause Mortality in an Emergency Department Population. CJC Open.

[18] Kavsak P.A., Cerasuolo J.O., Hewitt M.K., Mondoux S.E., Perez R., Seow H., Ainsworth C., Ma J., Worster A., Ko D.T. Identifying very low risk patients for future myocardial infarction or death. Can. J. Cardiol..

[19] Lu P., Lu X., Li B., Wang C., Wang X., Ji Y., Liu Z., Li X., Yi C., Song M. (2022). High-Sensitivity Cardiac Troponin T in Prediction and Diagnosis of Early Postoperative Hypoxemia after Off-Pump Coronary Artery Bypass Grafting. J. Cardiovasc. Dev. Dis..

[20] Devereaux P.J., Lamy A., Chan M.T.V., Allard R.V., Lomivorotov V.V., Landoni G., Zheng H., Paparella D., McGillion M.H., Belley-Côté E.P. (2022). High-Sensitivity Troponin I after Cardiac Surgery and 30-Day Mortality. N. Engl. J. Med..

[21] Thygesen K., Alpert J.S., Jaffe A.S., Chaitman B.R., Bax J.J., Morrow D.A., White H.D. (2018). Executive Group on behalf of the Joint European Society of Cardiology (ESC)/American College of Cardiology (ACC)/American Heart Association (AHA)/World Heart Federation (WHF) Task Force for the Universal Definition of Myocardial Infarction. Circulation.

[22] Zhang X., Sun Y., Zhang Y., Fang F., Liu J., Xia Y., Liu Y. (2022). Cardiac Biomarkers for the Detection and Management of Cancer Therapy-Related Cardiovascular Toxicity. J. Cardiovasc. Dev. Dis..

[23] Morfino P., Aimo A., Castiglione V., Vergaro G., Emdin M., Clerico A. (2022). Biomarkers of HFpEF: Natriuretic Peptides, High-Sensitivity Troponins and Beyond. J. Cardiovasc. Dev. Dis..

[24] Graziano F., Juhasz V., Brunetti G., Cipriani A., Szabo L., Merkely B., Corrado D., D’Ascenzi F., Vago H., Zorzi A. (2022). May Strenuous Endurance Sports Activity Damage the Cardiovascular System of Healthy Athletes? A Narrative Review. J. Cardiovasc. Dev. Dis..

[25] Costache A.-D., Leon-Constantin M.-M., Roca M., Maștaleru A., Anghel R.-C., Zota I.-M., Drugescu A., Costache I.-I., Chetran A., Moisă Ș.-M. (2022). Cardiac Biomarkers in Sports Cardiology. J. Cardiovasc. Dev. Dis..

